# Occurrence and distribution of pepper veinal mottle virus and cucumber mosaic virus in pepper in Ibadan, Nigeria

**DOI:** 10.1186/1743-422X-9-79

**Published:** 2012-04-11

**Authors:** Olawale Arogundade, Olusegun Samuel Balogun, Kehinde Titilope Kareem

**Affiliations:** 1 National Horticultural Research Institute, Idi Ishin, Jericho Reservation Area, P.M.B 5432, Dugbe, Ibadan, Oyo State, Nigeria; 2 University of Ilorin, Faculty of Agriculture, Crop Protection Department, P.M.B 1515, Ilorin, Kwara State, Nigeria

**Keywords:** Antisera, CMV, Diagnostic survey, ELISA, PVMV, Nigeria

## Abstract

Viral diseases constitute obstacles to pepper production in the world. In Nigeria, pepper plants are primarily affected by pepper veinal mottle virus (PVMV), Cucumber mosaic virus (CMV), Pepper leaf curl Virus (TLCV), Tobacco mosaic virus (TMV), Pepper mottle virus (PMV) and a host of other viruses. The experiment was carried out with a diagnostic survey on the experimental field of the National Horticultural Research Institute, Ibadan, Nigeria and on pepper farms in six local government areas within Ibadan Oyo State, Nigeria, forty samples were collected from each of the farms. Diseased samples were obtained from the field and taken to the laboratory for indexing. In ELISA test some of the samples from the pepper farms showed positive reaction to single infection with PVMV (36.79%), CMV (22.14%) while some others showed positive reaction to mixed infection of the two viruses (10%) but some also negative reaction to PVMV and CMV antisera (31.07).

## Introduction

Plant virus diseases cause major losses to agricultural crops around the world. Chemical agents similar to fungicides and bactericides are not effective in controlling virus diseases. Strategies for virus management in plant are mostly aimed at eradicating the source of infection to prevent it from reaching the crop and interfering with the movement of vectors in order to prevent the spread of the disease. However, the most effective means of controlling virus diseases is through cultivating the virus-resistant varieties.

Generally, viral infection causes visible symptoms such as various forms of mosaic and distortions in plants with consequent reductions in crop growth and yield. While reduction in plant size is the most general symptom induced by virus infection, there is probably some stunting of growth even with 'masked' or 'latent' infections, where the systemically infected plant shows no obvious sign of disease [1]. In nature, higher plants are commonly co-infected with multiple viruses and a number of disease syndromes are caused by interaction of two independent viruses. The accumulation dynamics of the interacting viruses in such mixed infection often change drastically [2]. Besides, mixed infections with two unrelated viruses, which are common in field plants, especially in tropical areas, often produce a more severe disease than that caused by either virus alone. For instance, tobacco on infection with potato virus × (PVX) and potato virus Y normally develop a more severe disease than that induced by either virus alone [3].

Pepper veinal mottle virus (PVMV) was first recognized as a distinct member of a group of viruses which was originally designated the *Potato virus Y *group but was later renamed the Potyvirus group [4]. PVMV occurs mainly in Africa, although it affects *Capsicum annuum *L. crops in Afghanistan [5] and India [6]. PVMV also occurs in *Capsicum *spp. in Sierra Leone and Zaire, [7]. PVMV has been reported in several West African countries, and in some parts of Nigeria [8,9]. There was a report that a strain of PVMV occurs naturally in *Telfairea occidentalis *(Cucurbitaceae) in Nigeria [10]. Strains of the virus are also experimentally transmissible to at least 35 species of the Solanaceae and to nine species of five other families (Aizoaceae, Amaranthaceae, Apocynaceae, Chenopodiaceae and Rutaceae) [11-14]. Symptoms expressed by the leaves of PVMV-infected plants include chlorosis of the veins, followed by systemic interveinal chlorosis, mottle, and small distortion of leaves and at times leaf abscission and fruit distortion occur [11]. There have been reports of one hundred percent losses of marketable pepper fruit due to infection with pepper viruses causing whole field to be abandoned prior to harvest and in some areas making cultivation of pepper to be uneconomical in some parts of Nigeria [8].

Cucumber mosaic virus (CMV) is worldwide in distribution. The virus causing cucumber mosaic has perhaps a wider range of host and attacks a greater variety of vegetables, ornamentals, weeds, and other plants than other viruses [15]. CMV is transmitted mainlyby the green peach aphid, *Myzus persicae*, and by *Aphis gossypii*, but it can be transmittedby other species of aphids [16].

In this paper we report the distribution of PVMV and CMV infection in pepper plants within Ibadan, Oyo state, Nigeria.

## Materials and methods

Survey for *Pepper veinal mottle virus*, genus Potyvirus, and *Cucumber mosaic virus*, genus Cucumovirus was conducted during the 2009 planting season in seven locations in Ibadan, Oyo State in the southwest agro-ecological zone of Nigeria.

The locations were the experimental field of the National Horticultural Research Institute (NIHORT), Lagelu Local government area, Akinyele Local government area, Egbeda Local government area, Ona Ara Local government area, Oluyole Local government area and Ido Local government area.

### Collection of diseased leaf samples

Diseased Leaf Samples were randomly collected from the experimental fields containing pepper in NIHORT while in the Local government areas two cultivated pepper farms were randomly surveyed per Local government. On each site 20 plants were randomly sampled from the population of plants on the field. Forty plants were sampled per location from cultivated pepper plants showing symptoms of mosaic, chlorosis, yellowing, stunting and mottle making a total of two hundred and eighty samples in all. The sampled leaves were then stored under Calcium chloride and were placed in the refrigerator prior to indexing.

### Virus detection

International Iinstitute of Tropical Agriculture (IITA) Virology laboratory modified protocols for Direct antigen coating (DAC - ELISA) was used for the detection of the presence of PVMV and CMV both from the forty infected pepper leaf samples collected per location. The PVMV and CMV antibodies used were AAB 328 antiserum diluted in the ratio 1:1000 and 1:3000 respectively with Phosphate Buffered Saline (PBS-T) (0.05% Tween 20: pH 7.4: 8.0 g NaCl, 0.2 g KH_2 _PO_4_, 1.1 g Na_2 _HPO_4 _0.2 g KCl, 0.2 g NaNO_3 _in 1 l H_2_O + 0.5 ml Tween 20 (0.05%)) from the Virology Laboratory of the International Institute of Tropical Agriculture (IITA) Ibadan.

### Viral indexing protocols

One hundred micro litre of antigen (e.g. sap) ground at 0.1 g of leaf sample in 1 ml of coating buffer was dispensed into each well of ELISA plate. The plate was then incubated at 37°C for 1 h and later washed three times with PBS-T after the incubation period. Cross adsorption was made by grinding 1 g of healthy pepper leaf in 20 mls of conjugate buffer conjugate buffer (1/2 PBS + 0.05% Tween 20 + 0.02% egg albumin + 0.2% PVP + 0.02 g NaN3). 100 μl of PVMV and CMV polyclonal (AAB 328) antisera diluted 1:1000 and 1:3000 in the adsorption solution was added to each of the ELISA plate and then incubated at 37°C for 1 h. After incubation the ELISA plate was washed three times with PBS-T. 100 μl of protein, A- alkaline phosphatase conjugate diluted in the ratio 1:15000 in conjugate buffer (1/2 PBS + 0.05% Tween 20 + 0.02% egg albumin + 0.2% PVP + 0.02 g NaN3) was added per well and the plate incubated at 37°C for 1 h. The plate was washed three times with PBS-T. 100 μl of 0.001 g/ml of p-nitrophenyl phosphate substrate in substrate buffer (97 ml diethanolamine + 800 ml H_2_O + 0.2 g NaNO_3 _add HCl to give pH 9.8) was added per well and incubated at room temperature for one hour. For all incubations plates were covered with ELISA cover plates to avoid edge effects and to maintain uniform temperature. Healthy pepper plants (*Capsicum *sp.) were used as negative control while PVMV and CMV infected *Capsicum *sp were used as positive control. After one hour the absorbance was measured at 405 nm using multiscan ELISA reader. The samples were considered positive when the ELISA reading exceeded that of the healthy control or was at least twice the reading for the healthy control.

## Results and discussion

**Figure 1:**
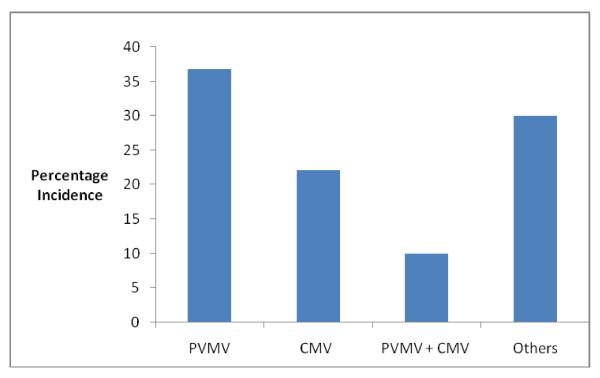
**Percentage of PVMV, CMV, PVMV + CMV and Other Virus diseases of pepper in Ibada**.

All the farms visited recorded the presence of PVMV and CMV as a single infection as well as mixed infection in some cases. Out of the two hundred and eighty leaf samples suspected to be virus infected collected from the farmers field in Ibadan, Oyo State of Nigeria 36.79% tested positive to PVMV, 22.14% tested positive to CMV, 10% tested positive to both PVMV and CMV through the serological test used, while 31.07% tested negative to the two antisera (Figure 1). However, from Table 1, out of the 40 leaf samples collected from each of the survey point Lagelu have the highest single infection of PVMV [3] while Ido have the highest single infection of CMV [12] while Egbeda have the highest incidence of mixed infection of PVMV and CMV [9].

**Table 1:** Distribution of PVMV and CMV in Ibadan

Location	Number of samples collected	PVMV	CMV	PVMV + CMV
NIHORT Experimental Field	40	17	10	6
Lagelu Local Government	40	19	10	0
Akinyele Local Government	40	14	7	4
Egbeda Local Government	40	11	8	8
Ona Ara Local Government	40	9	6	7
Oluyole Local Government	40	15	4	5
Ido Local Government	40	18	13	4

**Figure 2:**
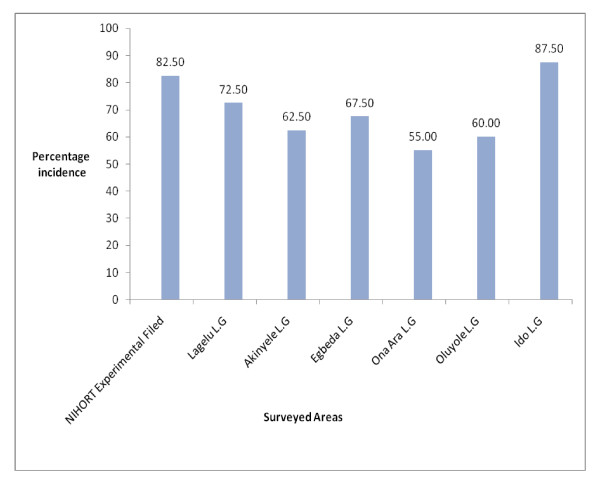
**Percentage Incidence of PVMV and CMV in NIHORT experimental Station and Six other areas of Oyo State**.

All the pepper farms surveyed within Ibadan showed differences in PVMV and CMV as observed in the percentage viral incidence (Figure 2). The percentage viral incidence of PVMV and CMV ranges between 55.0% in Ona-ara to 87.50% in Ido.

Pepper is highly susceptible to virus diseases in Ibadan, Oyo state, Nigeria as all samples picked from all the surveyed locations showed different viral symptoms ranging from different degree of mosaic, mottle, yellowing, stunting, puckering and in some cases reduction in leaf size which was similar to the earlier described symptoms of PVMV and CMV by Atiri [17] and Arogundade et al. [18]. It was however observed that from all the six local governments used as study area in this survey and the experimental field of NIHORT, pepper is mostly infected with PVMV than CMV and the mixed infection of PVMV and CMV, however the combine effect of PVMV and CMV is more on pepper than all other vegetable viruses combine as observed during the survey (Figure 1).

The high percentage incidence of PVMV and CMV in the experimental field of NIHORT could be as a result of numerous alternative host species surrounding the pepper field such as tomato, solanum, cucurbits and a host of other vegetables. This corroborates the findings of Alegbejo [19] who reported that the proximity of pepper plants to certain important weed hosts also has contributed greatly to the spread of viral diseases of pepper. These weeds include *Solanum nigrum, S. gracil, Physalis angulata, Vigna rosea, Vigna sinensis, Commelina nudiflora, Petunia hybrida, Physalis floridana, P. micrantha *and *Solanum incanum*.
